# Electroacupuncture Alleviates Experimental Chronic Inflammatory Pain by Inhibiting Calcium Voltage-Gated Channel-Mediated Inflammation

**DOI:** 10.1155/2020/7061972

**Published:** 2020-02-10

**Authors:** Jie Zhou, Ying Jin, Ruijie Ma, Hongyun Song, Qin Chen, Yueyang Chai, Yi Liang, You Zhou, Jianqiao Fang

**Affiliations:** ^1^The Third Affiliated Hospital of Zhejiang Chinese Medical University, 219 Moganshan Road, Xihu District, Hangzhou City, Zhejiang Province 310005, China; ^2^Department of Rehabilitation in Traditional Chinese Medicine, The Second Affiliated Hospital of Zhejiang University School of Medicine, No. 88, JiefangRd, Hangzhou City, Zhejiang Province 310000, China; ^3^Department of Emergency Medicine, The Second Affiliated Hospital of Zhejiang University School of Medicine, No. 88, JiefangRd, Hangzhou City, Zhejiang Province 310000, China; ^4^The Third Clinical Medical College, Zhejiang Chinese Medical University, 548 Binwen Road, Binjiang District, Hangzhou City, Zhejiang Province 310053, China; ^5^The Third Clinical Medical College, Zhejiang Chinese Medical University, Key Laboratory of Acupuncture and Neurology of Zhejiang Province, Hangzhou 310053, China

## Abstract

**Background:**

Both experimental and clinical studies have shown that electroacupuncture (EA) administration ameliorates chronic inflammatory pain (CIP). However, the multifaceted mechanism underlying the effects of EA on CIP is poorly understood. In this study, the mRNA transcriptome was used to study various therapeutic targets of EA.

**Methods:**

Using RNA-sequencing, protein-coding mRNA expression profiles of the L4-L5 dorsal root ganglion (DRG) were examined in the control (CN), complete Freund's adjuvant- (CFA-) induced CIP, and EA-treated CIP groups. A series of bioinformatics analyses was performed; “EA-reversed upregulated genes with CIP” (up-DEGs) and “EA-reversed downregulated genes with CIP” (down-DEGs) were identified. Thereafter, based on up-DEGs and down-DEGs, biological functions and signaling pathways were enriched using gene ontology (GO) and Kyoto encyclopedia of genes and genomes (KEGG) pathway analyses.

**Results:**

In total, 189 DEGs were identified, including 134 up- and 55 down-DEGs, which were enriched in arachidonic acid metabolism (rno00590), glutamatergic synapse (rno04724), serotonergic synapse (rno04726), FoxO signaling pathway (rno04068), insulin signaling pathway (rno04910), amyotrophic lateral sclerosis (rno05014), cholinergic synapse (rno04725), ECM-receptor interaction (rno04512), and choline metabolism in cancer (rno05231).

**Conclusion:**

We identified a few GOs, pathways, and genes that could play key roles in the amelioration of CIP by EA. Hence, this study may provide a theoretical basis for CIP amelioration by EA.

## 1. Introduction

Chronic inflammatory pain (CIP) is a type of refractory disease and occurs after peripheral nerve injury and tissue inflammation. Abundant studies have concluded that chronic pain affects countless individuals worldwide and has become a major public health concern, especially in elderly people [1, 2]. The incidence rate has reached approximately 33% in the general adult population and 56% in the elderly population, particularly in developing countries [3]. Hence, treatment methods to alleviate CIP are required.

Electroacupuncture (EA) is a commonly used acupuncture method and has been widely used to relieve CIP [4]. Previous studies on EA analgesia have mainly focused on the spinal and supraspinatus mechanisms; however, some studies have shown that local acupuncture had a better analgesic effect on chronic pain than distal acupuncture did [5]. Nociceptive information transferred from the periphery to the spinal cord can be modulated in peripheral sensory neurons, especially in the dorsal root ganglia (DRG), through the activation of the opioid system [6, 7]. Previous studies have shown that treatment with dilated 100 Hz and 2 Hz alternating frequency (2/100 Hz) EA at ST36 [8] can reduce the noxious response to complete Freund's adjuvant (CFA) stimulation, which is related to the peripheral endogenous opioid system and ERK1/2 and TRPV1 pathways [9–11]. Although both experimental and clinical studies have shown that electroacupuncture (EA) administration ameliorates chronic inflammatory pain (CIP), the multifaceted mechanism of EA for CIP is poorly understood.

Hence, to comprehensively elucidate the potential molecular mechanisms, our present study applied an RNA sequencing (RNA-seq) strategy to characterize the key molecular mechanisms of EA in CIP. First, we performed RNA-seq in the rat L4-5 DRG of the control, CIP, and EA groups and edgeR, and venny was applied to identify “EA-reversed upregulated genes with CIP” (up-DEGs) and “EA-reversed downregulated genes with CIP” (down-DEGs). Immediately after clusterProfiler, GO and pathway annotation of up-DEGs and down-DEGs were performed, respectively. Using quantitative PCR (qPCR) and western blotting (WB), we further demonstrated that the MAPK pathway induces inflammatory responses.

## 2. Materials and Methods

### 2.1. Animal Experiments

For animal experiments, we used a previously described procedure [10]. Briefly, 30 male Sprague Dawley (SD) rats (220–240 g, Certification no. SCXK 2014-0016) were purchased from Shanghai Laboratory Animal Center, Chinese Academy of Sciences. Animals were randomly divided into 5 groups (*n* = 6): (1) control group (*N*), (2) CFA group (*M*), (3) CFA + 100 Hz EA group (100 Hz), (4) CFA + 120 Hz EA group (120 Hz), and (5) CFA + 2/120 Hz EA group (2/120 Hz). The control group received a subcutaneous injection of 0.1 ml saline; the CFA group received a subcutaneous injection of 0.1 ml CFA and were immobilized; all three EA groups received a subcutaneous injection of 0.1 ml CFA, and were immobilized with EA treatment. All procedures strictly followed the National Institutions of Health Guide for the Care and Use of Laboratory Animals. The chronic inflammatory pain model was established by subcutaneously injecting 0.1 ml CFA (F5881-10ML, Sigma, USA) into the right hind paws of the rats.

### 2.2. EA Treatment

We performed the EA treatment as described in our previous study [9–11]. Rats in the CIP and EA group were immobilized using made-to-order cloth sleeves. However, only rats in the EA group received EA treatment. Two stainless steel acupuncture needles with a diameter of 0.25 mm were inserted into the right “Zusanli” (ST36) and “Kunlun” (BL60) acupoints. The two acupuncture needles were connected to the output of the Master-9 Pulse Stimulator (AMPI, Israel). The EA parameters were set as follows: bidirectional symmetric square wave; intensity range of 1-2 mA (15 min per intensity level, total 30 min); and the EA groups were subjected to EA with alternating frequencies and pulse waves. 100 Hz EA is characterized by a bidirectional rectangular wave with a 0.2 ms pulse width; 120 Hz EA is characterized by a bidirectional rectangular wave with a 0.3 ms width; and 2/120 Hz EA is a combination of 2 Hz EA and 120 Hz EA and was administered by alternately administering 2 Hz and 120 Hz 3 s each. EA was administered 30 min/day (1 mA for 15 min and 2 mA for 15 min) from day 1 after CFA injection for 10 consecutive days.

### 2.3. Inflammatory Cytokines Test

The rats were sacrificed at the end of EA treatment, and plantar tissues were collected. The collected plantar skin tissue was separated, and then tissue protein extraction was performed by using 100 mg tissue/ml PBS. The 0.4 M NaCl, tween 20 (0.05%), and protease inhibitors were also added along with PBS. The prepared sample was finally centrifuged for 10 min and the levels of inflammatory cytokines were analyzed, including IL-1*β* and TNF-*α*. Enzyme-linked immunosorbent assay (ELISA) was used to analyze the prepared sample using an ELISA kit (CUSABIO, Inc., Wuhan China).

### 2.4. Total RNA Preparation and Qualification

The L4-5 DRGs of all the rats were harvested after 10 days from the start of EA treatment. Total RNA from the harvested L4-5 DRGs was isolated and purified using TRIzol reagent (Invitrogen, Carlsbad, CA, USA) following the manufacturer's instructions. The quantity and quality of the RNA samples were determined using a NanoDrop 2000 (Thermo, Wilmington, DE, USA) and Agilent 2100 Bioanalyzer (Agilent Technologies, California, USA).

### 2.5. Library Construction and RNA Sequencing

We used the TruSeq® Stranded Total RNA Sample Preparation kit to prepare libraries following the manufacturer's instructions and quantified the purified libraries using a Qubit® 2.0 Fluorometer (Invitrogen, Life-Technologies, USA) and Agilent 2100 bioanalyzer (Agilent Technologies). A Cluster was generated by cBot with the library and sequenced on the Illumina HiSeq 2500 (Illumina, San Diego, CA). The sequencing was performed at Origin-Biotech Inc (Ao-Ji Biotech, Shanghai, China).

### 2.6. Bioinformatics Analysis

FastQC was conducted for Quality control (QC) of RNA-Seq reads (v. 0.11.3) (http://www.bioinformatics.babraham.ac.uk/projects/fastqc). Trimming was performed by seqtk for known Illumina TruSeq adapter sequences, poor reads, and ribosome RNA reads (https://github.com/lh3/seqtk). The trimmed reads were then mapped to the *Rattus norvegicus* reference genome (Ensembl genome browser: Rnor_6.0.91) by the Hisat2 (version: 2.0.4) [12, 13]. Stringtie (version: 1.3.0) was performed for each gene count from trimmed reads [13, 14]. Gene counts were normalized by trimmed mean of M-values [15], and fragments per kilobase of transcript per million mapped reads in Perl script [16]. EdgeR was performed to determine differential expression genes [17, 18] and threshold with *p* value < 0.05 and fold change >1.2. Venny was used to screen up-DEGs (CIP vs. control and EA vs. CIP) and down-DEGs (CIP vs. control and EA vs. CIP).

### 2.7. Functional Enrichment Analysis

GO and Kyoto Encyclopedia of Genes and Genomes (KEGG) pathways were enriched using R package to understand the functions of the DEGs better. In our study, clusterProfiler was applied to analysis of GO terms and KEGG pathways, and top30 GOs and pathways were presented [19, 20].

### 2.8. RNA Extraction and Real-Time PCR

Total RNA was extracted using Trizol reagent (Invitrogen, Carlsbad, CA, USA). cDNA was synthesized from total RNA using Transcriptor Reverse Transcriptase (Roche Applied Sciences, Indianapolis, IN, USA). Cacna1e (forward primer: 5′-ATGACGGGATCACCCAGTTT-3′; reverse primer: 5′-CAGTTCCAGGTGGCTCCTAA-3′) and Cacng5 (forward primer: 5′-ATCGGGTTCATCCTGAGCAA-3′; reverse primer: 5′-CAAGAGAGAGGCCGGATAGG-3′) genes were amplified with GAPDH (forward primer: 5′-AAGATGGTGAAGGTCGGTGT-3′; reverse primer: 5′-GCTTCCCATTCTCAGCCTTG-3′) as an internal control.

### 2.9. Western Blot

Total protein was extracted from L4-5 DRG neurons. We separated 30 *μ*g of protein from each sample on 12% SDS-PAGE and transferred the separated protein onto nitrocellulose membranes. Blots were first immunostained with primary antibody (Cacna1e: ab230640, Abcam, UK; Cacng5: ab96713, Abcam, UK; and GAPDH: ab181602, Abcam, UK) and subsequently with secondary antibody (CST anti-rabbit IgG, horseradish peroxidase-linked antibody CST 7074). GAPDH was used as a loading control.

### 2.10. Statistical Analyses

Descriptive data were expressed as mean ± standard deviation (SD) and compared between groups using one-way analysis of variance (ANOVA). In addition, where significant differences (*p* < 0.05) among groups were detected, specific group comparisons were made by least significant difference (LSD) tests. A *p* value of less than 0.05 was considered statistically significant. Statistical calculations were carried out using GraphPad Prism 8 (GraphPad Software, La Jolla, CA, United States).

## 3. Results

### 3.1. Effects of Electroacupuncture on the Expression of IL-1*β* and TNF-*α* in Plantar Tissue of CFA Rats

**Figure 1:**
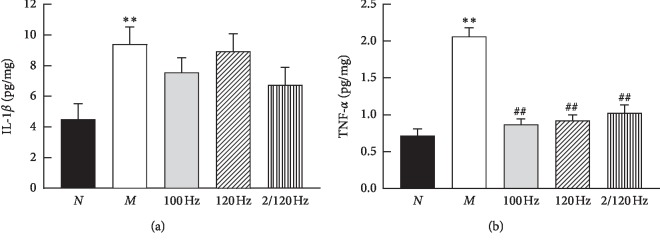
TNF-*α* and IL-1*β* levels in the plantar tissue in the five groups as detected by ELISA. *N* = control group, *M* = CFA group, 100 Hz = CFA + 100 Hz EA group, 120 Hz = CFA + 120 Hz EA group, 2/120 Hz = CFA + 2/120 Hz EA group. Statistical analysis was performed with one-way analysis of variance (ANOVA) and least significant difference (LSD) tests by GraphPad prism 8. Compared with N, ^*∗∗*^*p* < 0.01, compared with M, ^##^*p* < 0.01.

There was severe redness and swelling on ipsilateral plantar of the rats after CFA injection through the CFA model of observation. ELISA analysis revealed that the levels of IL-1*β* in the plantar tissue were significantly higher in the CFA-treated rats compared with the control group 24 h after injection (*p* < 0.01) (Figure 1). The levels of IL-1*β* in the plantar tissue of the EA rats were decreased in the EA groups compared with those in the CIP group; however, there was no significant difference (*p* > 0.05).

ELISA analysis also showed that the levels of TNF-*α* in the plantar tissue were significantly higher in the CFA-treated rats than in the controls (*p* < 0.01) (Figure 1). The levels of TNF-*α* in the plantar tissue of the EA rats was decreased in the EA groups (*p* < 0.01) compared with those in the CIP group; however, there was no significant difference between the EA groups (*p* > 0.05).

Based on the abovementioned results, we chose 2/120 Hz EA group as the representative to participate in the following experiments as we found that there was no significant difference between the EA groups (*p* > 0.05).

### 3.2. RNA-Seq Quality Assessment

After quality assessment of the sequences using seqtk, more than 33 million total original reads for each sample were obtained, and the proportion of bases with quality values greater than 20 (Q20) was more than 96%. These results indicated that the quality of the sequencing results was acceptable (Table 1). After filtering out the adaptor sequence and low quality reads, the percentage of clean reads within the raw reads accounted for 94% of the total sequences in four groups. The high proportion of clean reads and low number of low-quality or adaptor sequences demonstrated the excellent quality of the sequencing, which laid the foundation for high quality subsequent information analysis. Tophat software was used to map the obtained clean reads to the *Rattus norvegicus* reference genome. As shown in Table 1, approximately 77.2% of the clean reads were mapped onto the reference genome.

### 3.3. Identification of DEGs

**Figure 2:**
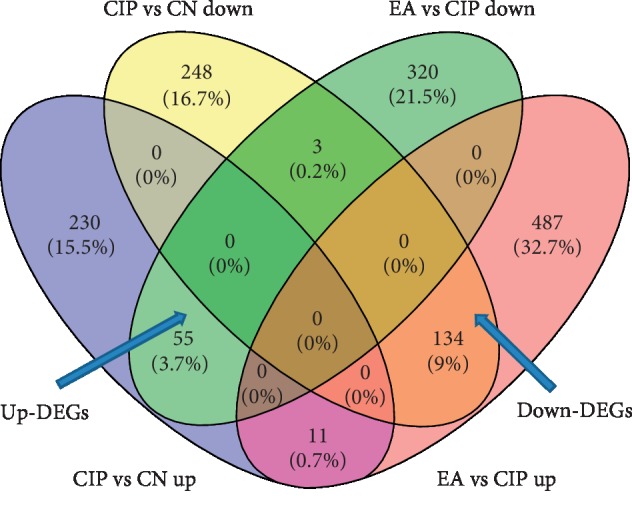
The Venn diagram of intersections of EA reversed dysregulated genes with CFA-induced CIP: (a) up-DEGs (55 up-DEGs were identified from the intersection of 296 up-regulated mRNAs (CIP vs. CN) and 378 up-regulated mRNAs (EA vs. CIP)) and (b) down-DEGs (134 down-DEGs were screened from the intersection of 385 down-regulated mRNAs (CIP vs. CN) and 632 up-regulated mRNAs (EA vs. CIP)).

**Figure 3:**
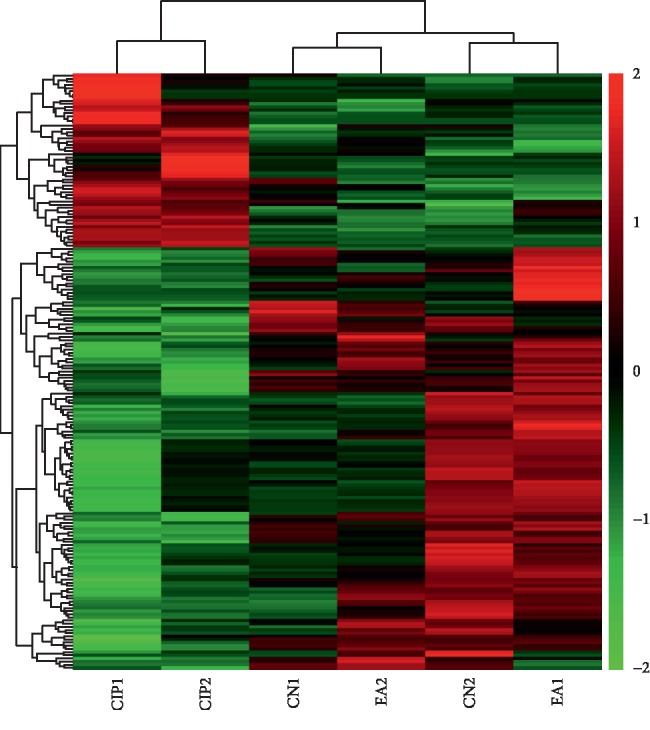
Heatmap of EA reversed down-DEGs and up-DEGS with total of 6 sampes (columns) and 189 genes (rows). CN1 and CN2 refer to the control group, CIP1 and CIP2 refer to the CFA-induced CIP group, and EA1 and EA2 refer to the EA treatment group. Red in the heatmap denotes upregulation, while green denotes downregulation.

To better understand the molecular mechanism of EA treatment of CIP, we performed RNA-seq of the transcriptome in the CN, CIP, and EA groups. We were able to identify 189 DEGs through bioinformatics analysis. As shown in Figure 2 and [Supplementary-material supplementary-material-1], 134 down-DEGs were screened from the intersection of 385 downregulated mRNAs (CIP vs. CN) and 632 upregulated mRNAs (EA vs. CIP) (Figure 2, [Supplementary-material supplementary-material-1]). On the other hand, 55 up-DEGs were identified from the intersection of 296 upregulated mRNAs (CIP vs. CN) and 378 upregulated mRNAs (EA vs. CIP) (Figure 2, [Supplementary-material supplementary-material-1]). A heatmap of the identified 189 DEGs is shown in Figure 3.

### 3.4. GO Analysis of DEGs

In order to further understand the genes associated with the mechanism through which EA relieves CIP, GO enrichment analysis was performed with 134 down-DEGs and 55 up-DEGS in clusterProfiler.

**Figure 4:**
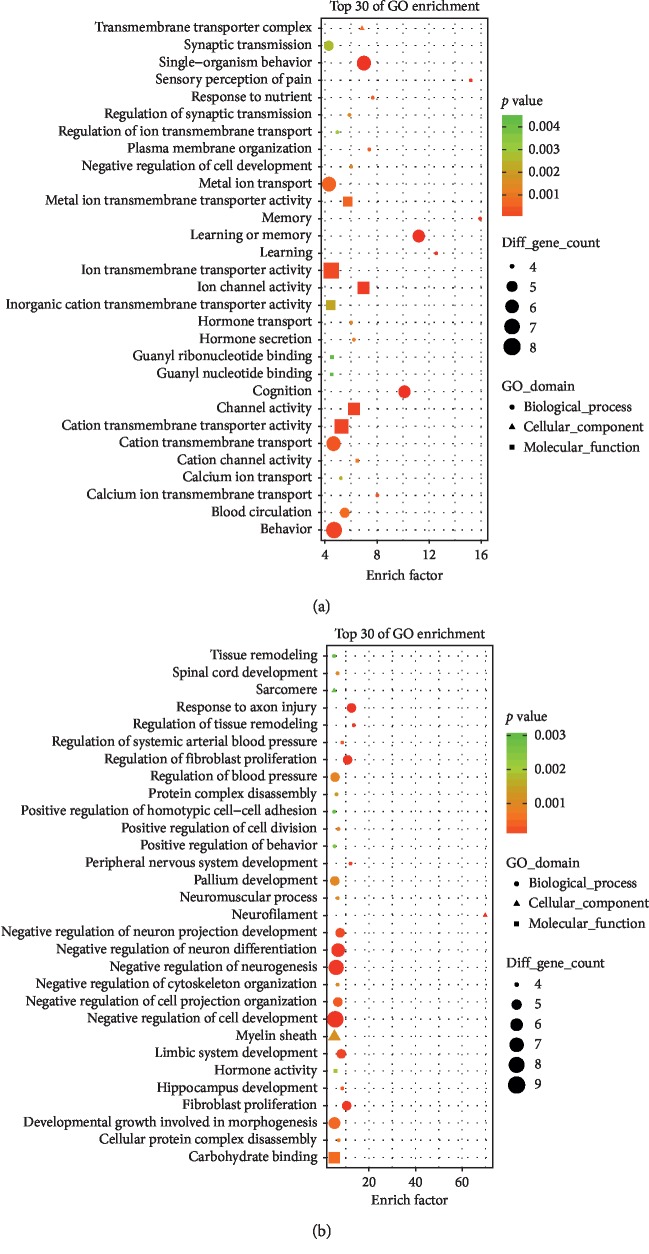
Gene ontology enrichment analysis for EA reversed up-DEGs and down-DEGs in CFA-induced CIP. The top 30 significant biological processes, molecular functions, and cellular components of up-DEGs (a) and the top 30 significant biological processes, molecular functions, and cellular components of down-DEGs (b). The dotted line indicated *p* value of 0.05.

Enrichment analysis showed that the 55 up-DEGs were significantly enriched in a total of 64 GO-terms; 45 were biological processes (BP), 6 were cellular components (CC), and 13 were molecular functions (MF) (*p* < 0.05) ([Supplementary-material supplementary-material-1]). Learning or memory (GO:0007611), cognition (GO:0050890), memory (GO:0007613), sensory perception of pain (GO:0019233), single-organism behavior (GO:0044708), learning (GO:0007612), behavior (GO:0007610), cation transport (GO:0006812), cell-cell signaling (GO:0007267), and cation transmembrane transport (GO:0098655) were the top 10 enriched GO BP terms. The significantly enriched CC terms were transmembrane transporter complex (GO:1902495), cell body (GO:0044297), plasma membrane protein complex (GO:0098797), cell junction (GO:0030054), cell projection (GO:0042995), and synapse (GO:0045202). The enriched GO:MF terms were ion channel activity (GO:0005216), cation channel activity (GO:0005261), channel activity (GO:0015267), metal ion transmembrane transporter activity (GO:0046873), cation transmembrane transporter activity (GO:0008324), guanyl ribonucleotide binding (GO:0032561), guanyl nucleotide binding (GO:0019001), ion transmembrane transporter activity (GO:0015075), inorganic cation transmembrane transporter activity (GO:0022890), and substrate-specific transmembrane transporter activity (GO:0022891). The top 30 GO-terms with highest enrichment factors are shown in Figure 4(a).

The 134 down-DEGs were significantly enriched in 252 GO-terms; 207 were BP, 31 were CC, and 14 were MF (*p* < 0.05) ([Supplementary-material supplementary-material-1]). The top 10 enriched GO:BP were cell projection organization (GO:0030030), response to axon injury (GO:0048678), neuron development (GO:0048666), behavior (GO:0007610), negative regulation of cell development (GO:0010721), regulation of cell projection organization (GO:0031344), regulation of fibroblast proliferation (GO:0048145), cell development (GO:0048468), neuron differentiation (GO:0030182), and fibroblast proliferation (GO:0048144). The top10 enriched GO : CC were neurofilament (GO:0005883), cell projection (GO:0042995), axon (GO:0030424), neuron projection (GO:0043005), cilium (GO:0005929), extracellular space (GO:0005615), myelin sheath (GO:0043209), somatodendritic compartment (GO:0036477), sarcomere (GO:0030017), and intermediate filament (GO:0005882). The top10 enriched GO:MF were carbohydrate binding (GO:0030246), cation transmembrane transporter activity (GO:0008324), hormone activity (GO:0005179), inorganic cation transmembrane transporter activity (GO:0022890), receptor binding (GO:0005102), ion transmembrane transporter activity (GO:0015075), metal ion transmembrane transporter activity (GO:0046873), transmembrane transporter activity (GO:0022857), substrate-specific transmembrane transporter activity (GO:0022891), and G-protein coupled receptor binding (GO:0001664). The top 30 GO-terms with highest enrichment factors are shown in Figure 4(b).

### 3.5. KEGG Analysis of DEGs

Pathway enrichment analysis provides a better understanding of the function of genes and their interaction. ClusterProfiler was applied to GO enrichment analysis with 134 down-DEGs and 55 up-DEGs, which were associated with EA treatment of CIP.

**Figure 5:**
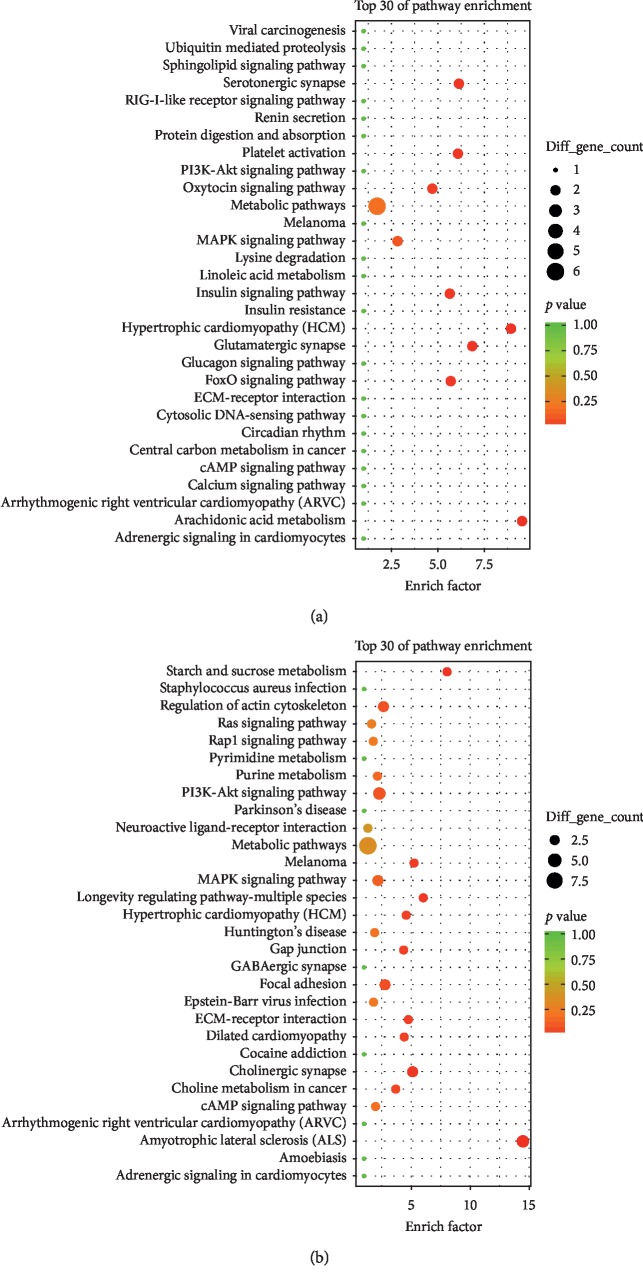
KEGG pathway enrichment analysis for EA reversed up-DEGs and down-DEGs in CFA-induced CIP. The top 30 significant KEGG pathway of up-DEGs (a) and the top30 significant KEGG pathway terms of down-DEGs (b). The dotted line indicated *p* value of 0.05.

KEGG enrichment analysis showed that 70 pathway terms, including 8 pathway terms, were enriched with 55 up-DEGs (*p* value <0.05) ([Supplementary-material supplementary-material-1]). The following are the pathways with *p* value <0.05: Arachidonic acid metabolism (rno00590), Hypertrophic cardiomyopathy (HCM) (rno05410), Glutamatergic synapse (rno04724), Serotonergic synapse (rno04726), Platelet activation (rno04611), FoxO signaling pathway (rno04068), Insulin signaling pathway (rno04910), and Oxytocin signaling pathway (rno04921). The top 30 pathway terms with the highest enrichment factors are shown in Figure 5(a).

KEGG enrichment analysis also showed that a total of 77 pathway terms were enriched with 134 down-DEGs ([Supplementary-material supplementary-material-1]), 10 of which were significant with *p* < 0.05, namely, Amyotrophic lateral sclerosis (ALS) (rno05014), Starch and sucrose metabolism (rno00500), Longevity regulating pathway - multiple species (rno04213), Melanoma (rno05218), Cholinergic synapse (rno04725), ECM-receptor interaction (rno04512), Hypertrophic cardiomyopathy (HCM) (rno05410), Dilated cardiomyopathy (rno05414), Gap junction (rno04540), and Choline metabolism in cancer (rno05231). The top 30 pathway terms with highest enrichment factors are shown in Figure 5(b).

### 3.6. Electroacupuncture-Inhibiting Calcium Voltage-Gated Channel with CIP

**Figure 6:**
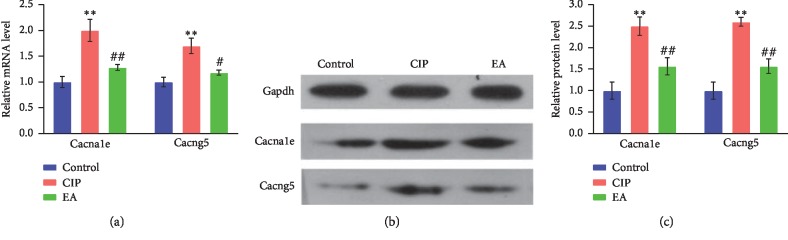
Cacna1e and Cacng5 mRNA (a) and protein (b, c) expression results obtained using qPCR and WB. Statistical analysis was performed with one-way analysis of variance (ANOVA) and least significant difference (LSD) tests by graphpad prism 8. Compared with control, ^*∗∗*^*p* < 0.01, compared with CIP, ^#^*p* < 0.01, ^##^*p* < 0.01.

RNA-seq analysis revealed that the calcium voltage-gated channel subunit (Cacna1e) and calcium voltage-gated channel auxiliary subunit gamma (Cacng5) are potentially involved in the EA regulation of CIP. For further confirmation, we performed qPCR and WB analysis of Cacna1e and Cacng5. As shown in Figure 6, Cacna1e mRNA and Cacng5 mRNA were significantly upregulated in the CIP groups compared with the control group with EA reversed the upregulation. These findings are consistent with those found in RNA-seq (Figure 6).

## 4. Discussion

IL-1*β* and TNF-*α* are major inflammatory chemokines and activators that are involved in mediating the generation and maintenance of pain [21]. Studies have shown that EA not only produces an analgesic effect but also inhibit peripheral and central inflammatory factors, such as IL-1*β*, TNF-*α*, PGE2, and COX-2 [22]. It was reported that EA significantly reduced the serum levels of TNF-*α* in adjuvant-induced arthritis (AA) rats [23]; researchers observed that EA could inhibit the secretion of IL-1*β* and TNF-*α* in osteoarthritis (OA) rats [24]; Wang et al. [25] revealed that EA could reduce TNF-*α* mRNA and protein expression, thus reducing tissue damage in neuropathic pain rats. In our study, we found that high-frequency and low-frequency EA stimulation downregulated the levels of IL-1*β* and TNF-*α* in CFA rats, which was consistent with findings from other studies on inflammatory pain [22, 26, 27].

We used high throughput RNA-seq to analyze the protein-coding mRNA expression profile in rat L4-5 DRG of the CN, CIP, and EA groups. Our results showed that EA reversed 189 genes with CIP, including 134 up-DEGs and 55 down-DEGs. GO and KEGG pathway analyses were performed to understand the mechanism of the EA effect on CIP better.

GO enrichment analysis revealed that EA can regulate dozens of neuron-related GO-BP terms, such as neuron projection, neuron fate commitment, neuron differentiation, neuromuscular process, neurogenesis, neuron apoptosis, regulation of neurotransmitter levels, neuron death, response to axon injury, axon guidance, axonogenesis, axon development, synaptic transmission, and postsynaptic membrane ([Supplementary-material supplementary-material-1]). In addition, GO-CC results showed EA could also affect neurofilament, neuron projection, neuronal cell body, somatodendritic compartment, dendrite, postsynaptic membrane, excitatory synapse, synaptic membrane, and synapse, axon. Therefore, it seems that the mechanism of EA against CIP involves many neuronal functions.

Some studies have reported that apoptosis is involved in neurotoxicity or pain [28–30], which coincides with our findings of significant enrichment of the negative regulation of the neuron apoptotic process (GO:0043524, *p* value = 1.212*e* − 02) and negative regulation of the apoptotic process (GO:0043066, *p* value = 1.628*e* − 02). Moreover, the FoxO signaling pathway (rno04068, *p* value = 4.735*e* − 02) and MAPK signaling pathway (rno04010, *p* value = 4.672*e* − 02) were also significantly enriched. Many studies have reported that the FoxO and MAPK signaling pathway can regulate apoptotic activity involved in pain or the nervous system [31–33]. Therefore, we speculate that EA can reverse the aberrant apoptotic activity in DRG neurons in CIP rats through the MAPK or/and signaling pathway.

In addition, we found significant enrichment of some neurotransmitter- and synapse-related GO-terms and pathway-terms, including Arachidonic acid metabolism (rno00590), Glutamatergic synapse (rno04724), Serotonergic synapse (rno04726), Cholinergic synapse (rno04725), and Choline metabolism in cancer (rno05231). Therefore, it seems that EA can repair the DRG neuron composition and activity that has been altered in CIP.

There are certain limitations in this study. First, it lacks the control group with only acupuncture, not electroacupuncture, for example, the control + acupuncture group. Second, a comparison with some conventional treatment group should be interesting. Last but not the least, in clinical practice, acupuncture points are selected dialectically according to different characteristics. Nonetheless, in order to standardization and methodological rigor, we adopt a mechanistic approach. However, the purpose of our research is studying the efficacy of electroacupuncture. Choosing dialectically according to different characteristics will be performed in our further study.

## 5. Conclusion

We analyzed the protein-coding mRNA expression profile in DRG neurons of rats in the CN, CIP, and EA groups using high throughput RNA-seq. We identified a number of genes, GO-terms, and pathway-terms that were involved in the treatment effect of EA on CIP. Taken together, these findings provide a clue toward understanding the underlying molecular mechanism of the treatment effect of EA on CIP.

## Figures and Tables

**Table 1:** Statistical data of the RNA-seq.

Sample	Total reads	Clean reads	Clean ratio (%)	No. rRNA pair	Mapped reads	Mapped ratio (%)
CN1	46684410	45838117	98.19	45115744	43256265	0.958784255
CN2	56691810	55731107	98.31	54774644	52505182	0.958567289
CIP1	44739180	43909611	98.15	43182178	41406951	0.958889823
CIP2	46747314	45973499	98.34	45277926	43375941	0.957993107
EA1	47413304	46609058	98.3	45890136	44028657	0.959436185
EA2	49317892	48519664	98.38	47805156	45892505	0.959990696

## Data Availability

All data generated or analyzed during this study are included in this published article. The raw data used and/or analyzed during the current study can be available from the corresponding author on reasonable request.

## Supplementary Materials

Supplementary Materials Supplement Tables. Table S1. The list of the 55 up-DEGs and 134 down-DEGs was identified for EA-revised genes with CIP. Table S2. The details of GO enrichment for the 55 EA-revised up-DEGs were identified for with CIP. Table S3. The details of GO enrichment for the 134 EA-revised down-DEGs were identified for with CIP. Table S4. The details of KEGG enrichment for the 55 EA-revised up-DEGs were identified for with CIP. Table S5. The details of KEGG enrichment for the 134 EA-revised down-DEGs were identified for with CIP.
